# Feasibility and Safety of the Left Distal Radial Approach in Percutaneous Coronary Intervention for Bifurcation Lesions

**DOI:** 10.3390/jcm10102204

**Published:** 2021-05-19

**Authors:** Oh-Hyun Lee, Ji Woong Roh, Eui Im, Deok-Kyu Cho, Myung Ho Jeong, Donghoon Choi, Yongcheol Kim

**Affiliations:** 1 Division of Cardiology, Department of Internal Medicine, Yonsei University College of Medicine and Cardiovascular Center, Yongin Severance Hospital, Yongin 16995, Korea; decenthyun@yuhs.ac (O.-H.L.); nomgalda@yuhs.ac (J.W.R.); imeui97@yuhs.ac (E.I.); chodk123@yuhs.ac (D.-K.C.); cdhlyj@yuhs.ac (D.C.); 2 Department of Cardiology, Chonnam National University Hospital, Gwangju 61469, Korea; myungho@chol.com

**Keywords:** radial artery, percutaneous coronary intervention, hematoma, distal radial approach

## Abstract

Recently, the left distal radial approach (DRA) for percutaneous coronary intervention (PCI) has been shown to be a feasible option, but there are limited data regarding the feasibility of performing bifurcation PCI via the left DRA. Therefore, this study aimed to describe our experience with the contemporary left DRA to perform PCI of bifurcation lesions. Between December 2017 and December 2019, we identified 106 patients treated with bifurcation PCI via the left DRA. We evaluated the success rate of PCI, access-site complications including major bleeding requiring surgery or transfusion, hematoma, distal and forearm radial artery occlusion, and 30-day mortality. Eleven patients (10.4%) treated with left main bifurcation and true bifurcations accounted for 39.6% of cases, with the left anterior descending artery/diagonal branch being the most frequent bifurcation site (57.5%, 61/106). PCI was performed using a 6-French guiding catheter in 101 (95.3%) cases. Successful PCI for bifurcation lesions via the left DRA was achieved in all 106 patients without access-site cross-over. There was no major bleeding, distal and forearm radial artery occlusion, forearm hematoma, or mortality at 30 days. The left DRA is a safe and feasible alternative access site for bifurcation PCI in selected patients.

## 1. Introduction

The conventional radial approach is widely accepted, and current guidelines recommend the radial approach as the standard approach for coronary angiography (CAG) and percutaneous coronary intervention (PCI) irrespective of clinical presentation [1,2]. Cardiac catheterization with the radial approach has been reported to be more beneficial than femoral approaches with easier hemostasis, better patient comfort, earlier ambulation, shorter hospital stays, and fewer occurrences of short-term cardiac death, myocardial infarction, and access-site complications [3].

Although previous studies have shown significantly lower rates of subclavian tortuosity and slightly shorter fluoroscopy times with the left radial approach than with the right radial approach, operators prefer to perform CAG or PCI via the right radial approach as the left radial approach is not as ergonomic for the operator, especially when treating obese patients [4]. In this situation, CAG or PCI with the left distal radial approach (DRA) in the anatomical snuffbox is an alternative approach to radial artery cannulation that does not cause the operator physical discomfort [5]. The left DRA can show advantages over the conventional radial approach such as reduction of radial artery occlusion [6,7].

Coronary bifurcation lesions are involved in 15–20% of all PCIs and remain one of the challenging lesions in terms of the procedural success rate and long-term cardiac events [8]. Previous studies have shown that the conventional radial approach is a feasible approach to the transfemoral approaches for non-left main and left main bifurcation PCI [9,10]. However, there is a paucity of data regarding the feasibility of performing PCI with the left DRA for bifurcation lesions. Therefore, this study aimed to describe our experience with the contemporary left DRA to perform PCI of bifurcation lesions.

## 2. Materials and Methods

### 2.1. Subjects and Study Design

**Figure 1:**
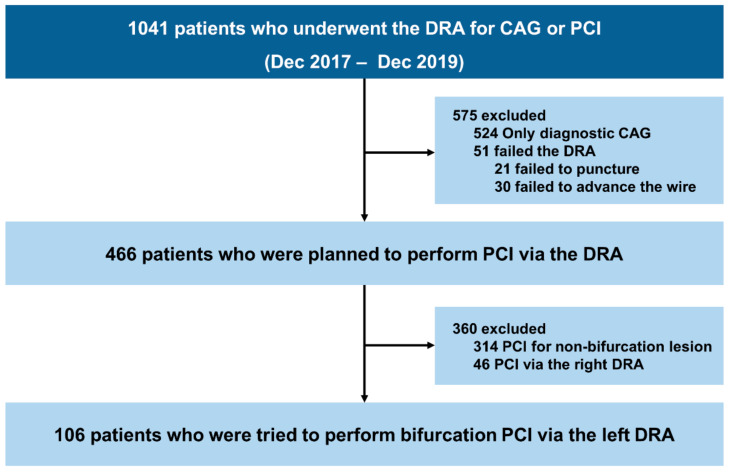
Study flowchart. Abbreviations: CAG, coronary artery angiography; DRA, distal radial approach; PCI, percutaneous coronary intervention.

We retrospectively collected data from patients who underwent PCI for bifurcation lesions via the left DRA at Chonnam National University Hospital between December 2017 and December 2019 (Figure 1). Coronary bifurcation lesions with a side branch (SB) diameter ≥2.5 mm according to quantitative coronary angiography were included. The exclusion criteria were as follows: (1) an SB diameter <2.5 mm, (2) patients who underwent PCI via the right DRA, and (3) non-bifurcated lesions. This study protocol was approved by the Institutional Review Board of Chonnam National University Hospital (approval number: CNUH-2021-046) and registered with the Clinical Research Information Service (https://cris.nih.go.kr/cris/en (Approved date: 8 April 2021, unique identifier: KCT0006072).

### 2.2. Preparation for the Left Distal Radial Approach

**Figure 2:**
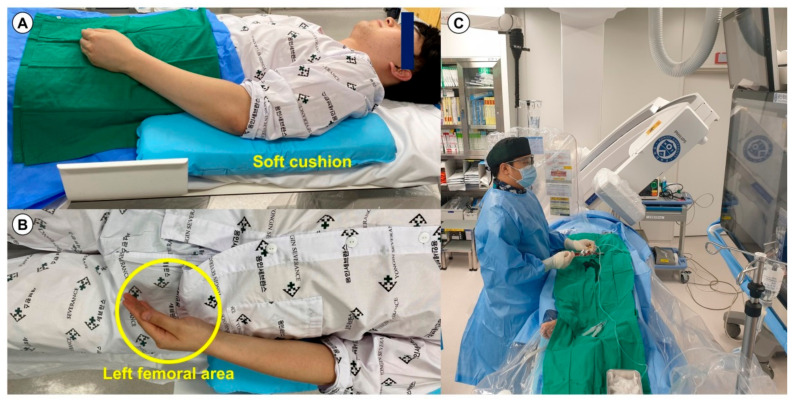
Positions of the patient and operator during the left DRA. The lateral (**A**) and front views (**B**) of the patient’s preparation position during the left DRA. (**C**) The natural working position of the operation while performing coronary angiography via the left DRA. Abbreviation: DRA, distal radial approach.

**Figure 3:**
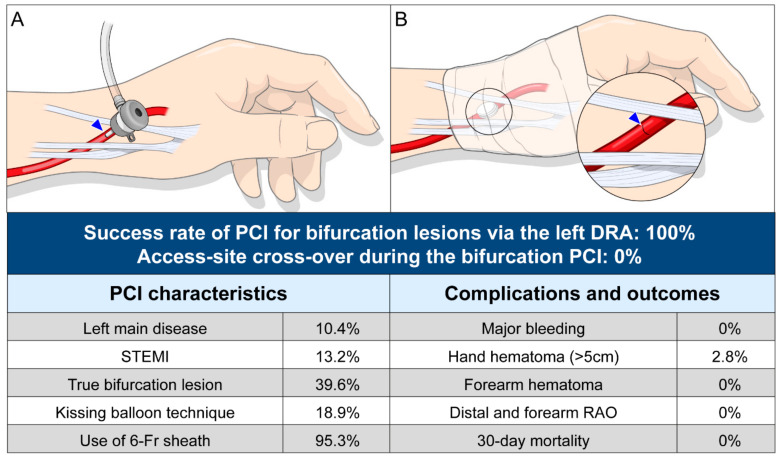
Inserted 6-Fr sheath via the left distal radial artery in the anatomical snuffbox area (blue arrowhead: puncture site) (**A**) and dressing for hemostasis with compressive bandage with gauze (**B**) and summary of the study outcomes. Abbreviations: DRA, distal radial approach; PCI, percutaneous coronary intervention, RAO, radial artery occlusion; STEMI, ST-elevation myocardial infarction; Fr, French.

All patients had a palpable arterial pulse in the anatomical snuffbox. The patient’s left hand was turned clockwise at 90°, allowing the snuffbox to face the ceiling, and it was slightly bent toward the patient’s body and placed on his/her left groin (Figure 2A,B). The operator prepared for the left DRA and performed coronary procedures on the right side of the patients (Figure 2C). The puncture site of the left distal radial artery was sterilized in all patients. Local anesthesia of the anatomical snuffbox was achieved through a 1-cc lidocaine hydrochloride injection. Thereafter, puncture was performed by using a 20-gauge two-piece needle or a 21-gauge open needle. After a successful puncture, a 0.025-inch straight wire or a 0.018-inch hair wire was inserted, followed by insertion of the 5- or 6-French (Fr) radial sheath (Prelude^®^ Radial, MERIT MEDICAL, South Jordan, UT, USA or Radiofocus^®^ Introducer II, TERUMO Corp., Tokyo, Japan) (Figure 3A and Video S1). After successful sheath insertion, 3000 U of unfractionated heparin, 200 μg of nitroglycerin, and 2 mg of verapamil were administered intrasheath to reduce the rate of spasm and post-procedural radial arterial occlusion. After completion of PCI, the radial sheath was removed, and a compression cohesive elastic bandage with a 4 × 4-inch sterile gauze dressing for hemostasis was applied for 3 h in all patients (Figure 3B).

### 2.3. Procedural Details of Percutaneous Coronary Intervention for Bifurcation Lesions

Following placement of a sheath introducer, coronary angiography was performed according to the standard technique. Additional unfractionated heparin (50–70 U/kg) was administered during the procedure to maintain the activated clotting time at 250–300 s. The treatment strategy for SB including balloon angioplasty or stenting, use of a coronary stent, use of intravascular modalities, and use of an additional device (e.g., a microcatheter and guide extension catheter) were determined at the operator’s discretion.

### 2.4. Definition and Study Endpoints

Bifurcation lesions were assessed according to the Medina classification, which classifies the lesions according to the angiographic involvement of the three relevant segments of the bifurcation—the proximal main vessel, distal main branch, and SB [11]. Lesions that include both the main vessel and the SB are considered a true bifurcation.

The primary endpoint was the successful rate of PCI for bifurcation lesions via the left DRA. The secondary endpoints were major bleeding requiring any transfusion or surgery, access-site complications including hematoma and forearm radial and/or distal radial artery occlusion, and 30-day mortality.

### 2.5. Statistical Analysis

Normally distributed continuous variables are expressed as mean ± standard deviation. Non-normally distributed continuous variables are reported as median and interquartile range. All categorical variables are presented as number (percentage). All statistical analyses were performed using SPSS statistical software (SPSS version 25.0 for Windows; IBM Corp., Armonk, NY, USA).

## 3. Results

Between December 2017 and December 2019, we identified 1041 patients who underwent the DRA for CAG or PCI. Among them, we selected 106 patients who underwent PCI via the left DRA for bifurcation lesions (Figure 1). Baseline characteristics of the study population are presented in Table 1. The overall mean age of the patients was 63.3 ± 11.1 years (range, 35–89 years), and 79.2% (84/106) of patients were male. Of the 95 (89.6%) patients presenting with acute coronary syndrome, 14 (13.2%) patients had ST-elevation myocardial infarction. The mean left ventricular ejection fraction was 61.3 ± 11.0%. All patients, including 29 (27.4%) patients taking a potent P2Y12 inhibitor (ticagrelor, 12.3% (13/106); prasugrel, 15.1% (16/106)), were treated with dual antiplatelet therapy.

The success rate of PCI for bifurcation lesions was 100% in all 106 patients, and no patients required cross-over to the conventional radial or femoral approaches. Angiographic and procedural characteristics are provided in Table 2. The overall success rate of DRA was 95.1% (990/1041). Regarding the details of the left DRA, the mean and median times of distal radial artery puncture, defined as the time interval from local anesthesia induction to successful sheath cannulation, were 2.4 ± 1.6 and 1.8 min (quartiles 1–3: 1.3–3.4), respectively. The distal radial artery puncture was performed within 3 and 5 min in 71.7% (76/106) and 93.4% (99/106) of patients, respectively. Concerning lesion characteristics, 11 (10.4%) patients were treated for left main bifurcation lesions via the left DRA, and true bifurcations accounted for 39.6% of cases, with the left anterior descending artery/diagonal branch being the most frequent bifurcation site (57.5%, 61/106). Four patients were treated with a planned two-stent technique (mini-crush two-stent technique). Twenty-six (24.5%) and four (3.8%) patients were treated with plain balloon angioplasty and stent implantation of the SB, respectively. The kissing balloon technique for bifurcated lesions was performed in 20 (18.9%) patients. Implantation of ≥2 stents was performed in 23 (21.7%) patients, and intravascular imaging-guided PCI was conducted in 25 (23.6%) patients (10, intravascular ultrasound; 15, optical coherence tomography guidance). Overall, 11.3% of patients underwent multi-vessel PCI. An extra-backup type catheter was used in 71 of 96 cases (74.0%) with a left coronary artery lesion. Among 18 cases of right coronary artery lesion, Judkins right and Amplatz type catheters were used in four (22.2%) and 14 (77.8%) cases, respectively. PCI was performed using a 6-Fr guiding catheter in 101 patients (95.3%). In two (1.9%) patients, a 7-Fr guiding catheter was used.

Regarding puncture-site complications, there were no major bleeding complications requiring surgery or transfusion. Hand hematoma occurred in five patients (4.7%) including three (2.8%) patients with a >5-cm-diameter hematoma, but no case of forearm hematoma was observed. There was no incidence of distal and forearm radial artery occlusion and death at 30 days (Table 3).

## 4. Discussion

The principal findings of the current study were as follows: (1) the success rate of the bifurcation PCI via the left DRA was high without the need for access-site cross-over; (2) the occurrence rate of puncture site complications was low (4.7%), and there was no major bleeding, distal and forearm radial artery occlusion, forearm hematoma, or mortality at 30 days. To the best of our knowledge, this is the first clinical study to investigate the feasibility of the left DRA for bifurcation PCI, which remains a challenge.

Previous studies on the left DRA are summarized in Table 4, and these studies have reported a 97.6–98.9% success rate of PCI via the left DRA, except for the study on PCI for chronic total occlusion via the left DRA [5,12,13,14,15,16]. In the current study, bifurcation PCI via the left DRA was successfully performed in all 106 patients without access-site cross-over. Therefore, our study reaffirms the high success rate of PCI via the left DRA and suggests that the left DRA could be feasible as an alternative access site for bifurcation PCI.

The right conventional radial approach is commonly used by most operators probably because catheter manipulation can be performed from the patient’s right side without physical discomfort of the operator. However, with the right conventional radial approach, anatomic variations such as an increased incidence of tortuosity and loops in the arm and subclavian arteries may require extra catheter manipulation, and backup force for the right coronary artery is poor due to the S-shaped geometry of the subclavian–innominate–aorta axis, which can increase procedural complexity [17]. Furthermore, during the right conventional radial approach, the catheter needs to be passed from the innominate artery into the ascending aorta where the right carotid artery comes off, resulting in an increased risk of embolic stroke [4]. By contrast, the left conventional radial approach offers a very similar technique to the femoral approach, which makes it easy to manipulate the catheter and reduce the fluoroscopic time, procedure time, contrast volume, radiation exposure, and access-site change [17,18,19]. In the present study, the strength of the left side approach allowed us to achieve the high success rates of bifurcation PCI. Physical discomfort of the operator is generally recognized as a barrier to the left conventional radial approach, especially if the patient is obese or the operator is short [20]. This barrier can be overcome by the left DRA, in which the patient’s elbow is slightly bent, and the left hand is positioned above the left groin. As Figure 2 shows, there is no excessive bending of the elbow, which causes difficulties in wire or guiding catheter advancement, or kinking of the guiding catheter near the elbow and the operator position is the same as that in the right conventional radial approach, which may have led to the high success rate of PCI for bifurcation lesions via the left DRA in the current study.

Indeed, there are concerns regarding PCI via the DRA because the diameter of the distal radial artery in the anatomical snuffbox was significantly smaller than that of the conventional radial artery [14]. However, our study showed that 95.3% (101/106) of patients underwent successful PCI using a 6-Fr guiding catheter without major puncture-site complications. Moreover, with the recent introduction of the thin-wall 7-Fr sheath (Prelude IDeal^TM^, MERIT MEDICAL; Glidesheath Slender^®^, TERUMO Corp.) or sheathless guiding catheter (Sheathless Eaucath, Asahi Intecc, Tokyo, Japan; Railway^TM^, Cordis Corporation, Miami, FL, USA) [15,21,22,23], complex PCI, including bifurcation PCI, via the left DRA can be more easily and safely performed in the relatively smaller diameter vessels. Colletti et al. reported the feasibility and safety of performing bifurcation PCI (75%, 15/20) with a 7-Fr sheathless system via the DRA in a limited number (*n* = 20) of study patients [21]. Further studies are needed to evaluate the feasibility of PCI using a sheathless guiding catheter or thin-wall sheath via the DRA in a large study population.

Previous studies regarding the feasibility of the left DRA in patients who underwent CAG and PCI, as summarized in Table 4, showed that major complications did not occur. It is noteworthy that the current study showed the safety of the left DRA with a small number of minor complications and no major complications, which is similar to the findings of previous studies, despite the use of a 6-Fr sheath in most patients. However, the majority of patients in the current study are male and young and may have a relatively large diameter of distal radial artery. These demographic findings may have affected the result. Further large-scale, well-designed studies regarding the safety of complex PCI via the left DRA are required.

Some limitations of our study should be noted. First, the study was a retrospective, non-randomized, observational study, which has inherent selection and information biases. Second, the small number of patients in the present study rendered low statistical power. Nevertheless, this is the first clinical study to investigate the feasibility of the left DRA for bifurcation PCI, which remains a challenge. Third, details of the technical option for bifurcation PCI such as the jailed technique for SB protection (e.g., the jailed-wire, jailed-balloon, or jailed Corsair technique) or the selected balloon for the kissing balloon technique were not considered. Fourth, although the overall success rate of the DRA is high at over 95% in the current study, we should consider that puncture failure still remains a hurdle in the DRA. Fifth, the majority of patients in this study are male and young, and they may have relatively large diameter distal radial arteries compared with elderly and female patients. Furthermore, patients who had highly complex bifurcation lesions, requiring a 7-Fr guiding catheter, may have been excluded because the majority of patients were treated using a 6-Fr guiding catheter in our study. In interpreting the results of the current study, the groups of subjects should not be expanded, and the results should be interpreted with caution. Sixth, the lack of a comparator group of patients undergoing the conventional radial approach is another potential limitation. Seventh, the lack of routine post-procedural ultrasonography for forearm and radial artery may have contributed to underestimation of the puncture-site complications. Eighth, we did not investigate long-term clinical outcomes after 30 days because the present study focused on the technical success.

## 5. Conclusions

PCI via the left DRA was a feasible option in selected patients with bifurcation lesions, and this was supported by the high PCI success rate without access-site cross-over. Regarding the safety aspect of PCI via the left DRA for bifurcation lesions, there were no significant access-site complications or adverse clinical outcomes. Further large randomized trials should be conducted to evaluate the feasibility of the left DRA for bifurcation PCI.

## Figures and Tables

**Table 1:** Baseline characteristics (*n* = 106).

Characteristic	Value
Age (years)	63.3 ± 11.1
Male sex	84 (79.2%)
Height (cm)	167.5 (160, 172)
Weight (kg)	69.0 ± 11.1
Body mass index (kg/m^2^)	25.0 ± 2.9
Hypertension	64 (60.4%)
Diabetes mellitus	33 (31.1%)
Dyslipidemia	54 (50.9%)
Current smoker	29 (27.4%)
Chronic kidney disease, ≥stage 3	15 (14.2%)
Clinical presentation	
Stable angina	11 (10.4%)
Unstable angina	54 (50.9%)
NSTEMI	27 (25.5%)
STEMI	14 (13.2%)
LVEF (%)	61.3 ± 11.0
Periprocedural medication	
DAPT	106 (100%)
Aspirin	106 (100%)
P2Y12 inhibitor	
Clopidogrel	77 (72.6%)
Ticagrelor	13 (12.3%)
Prasugrel	16 (15.1%)
Oral anticoagulation	3 (2.8%)
Glycoprotein IIb/IIIa inhibitors	5 (4.7%)

Data are presented as mean ± standard deviation, median (interquartile range), or number (%). Abbreviations: DAPT, dual antiplatelet therapy; LVEF, left ventricular ejection fraction; NSTEMI, non-ST-elevation myocardial infarction; STEMI, ST-elevation myocardial infarction.

**Table 2:** Angiographic and procedural characteristics (*n* = 106).

Characteristic	Value
Successful bifurcation PCI via the left DRA	106 (100%)
Cross-over to another vascular approach	0 (0)
Characteristics of the left DRA	
Puncture time (min)	
Mean ± SD	2.4 ± 1.6
Median (IQR)	1.8 (1.3, 3.4)
Puncture time <3 min	76 (71.7%)
Puncture time <5 min	99 (93.4%)
Lesion characteristics	
Target lesion	
Left main coronary artery	11 (10.4%)
Left anterior descending artery	61 (57.5%)
Left circumflex artery	18 (17.0%)
Right coronary artery	16 (15.1%)
Medina classification	
0, 0, 1	2 (1.9%)
0, 1, 0	21 (19.8%)
0, 1, 1	9 (8.5%)
1, 0, 0	16 (15.1%)
1, 0, 1	9 (8.5%)
1, 1, 0	25 (23.6%)
1, 1, 1	24 (22.6%)
True bifurcation	42 (39.6%)
ACC/AHA type B2/C lesion	57 (53.8%)
Procedural characteristics	
Treatment of the side branch	
Plain balloon angioplasty	26 (24.5%)
Stenting (two-stent technique)	4 (3.8%)
Kissing balloon technique	20 (18.9%)
Intravascular imaging-guided PCI	25 (23.6%)
IVUS guidance	10 (9.4%)
OCT guidance	15 (14.2%)
Multi-vessel PCI	12 (11.3%)
Total no. of implanted stents	1.26 ± 0.54
Cases with implantation of ≥2 stents	23 (21.7%)
Stent diameter in the main vessel (mm)	2.98 ± 0.38
Total stent length in the main vessel (mm)	31.8 ± 14.3
Left guiding catheter (*n* = 96)	
EBU type catheter	71 (74.0%)
Judkins left type catheter	25 (26.0%)
Right guiding catheter (*n* = 18)	
Judkins right type catheter	4 (22.2%)
Amplatz type catheter	14 (77.8%)
Guiding catheter size	
5-Fr	3 (2.8%)
6-Fr	101 (95.3%)
7-Fr	2 (1.9%)

Data are presented as mean ± SD, median (interquartile range), or number (%). Abbreviations: ACC, American College of Cardiology; AHA, American Heart Association; DRA, distal radial approach; EBU, extra-backup; IVUS, intravascular ultrasound; IQR, interquartile range; OCT, optical coherence tomography; PCI, percutaneous coronary intervention; SD, standard deviation; Fr, French; no., number.

**Table 3:** Safety outcomes.

Variable	Total Patients (*n* = 106)
30-day mortality	0
Any bleeding complication requiring surgery or transfusion	0
Access-site complication	
Distal radial artery occlusion	0
Forearm radial artery occlusion	0
Hand hematoma	5 (4.7%)
≤5 cm in diameter	2 (1.9%)
>5 cm in diameter	3 (2.8%)
Forearm hematoma	0

Data are presented as number (%).

**Table 4:** Summary of previous articles regarding the left DRA.

Study, Author Name (y of Publication)	No. of Patients	Mean Age (y)	Male Sex	Left DRA	Success Rate of the Puncture Site	PCI	Bifurcation	Success Rate of PCI	Cross-over in PCI	Distal RAO	Major Bleeding Requiring Transfusion or Surgery
Kiemeneji et al. (2017) [5]	70	68 ± 11	55 (79%)	100%	89%	25 (36%)	N/A	N/A	1	1 (1.5%)	0
Kim et al. (2018) [14]	150	66 ± 13	94 (71%)	100%	88%	42 (28%)	N/A	97.6%	1	N/A	0
Lee et al. (2018) [16]	200	66 ± 12	132 (66%)	100%	95.5%	87 (44%)	28/87 (32.2%)	98.9%	0	0	0
Soydan et al. (2018) [12]	54	59 ± 12	43 (80%)	100%	100%	20 (37%)	N/A	N/A	2	0	0
Al-Azizi et al. (2018) [13]	61	70	46 (75%)	100%	98.4	34 (56%)	N/A	N/A	0	0	0
Gasparini et al. (2019) [15]	41	68 ± 5	31 (76%)	100%	82.9%	100%	N/A	78.1%	N/A	1 (4.3%)	0

Data are presented as number (%). Abbreviations: DRA, distal radial approach; PCI, percutaneous coronary intervention; RAO, radial artery occlusion; no., number; N/A, not applicable.

## Data Availability

The underlying data set is available from the corresponding author upon reasonable request.

## Supplementary Materials

The following are available online at https://www.mdpi.com/article/10.3390/jcm10102204/s1, Video S1: How to perform left distal radial artery puncture.
